# Spatio-Temporal Variation in Nutrient Profiles and Exchange Fluxes at the Sediment-Water Interface in Yuqiao Reservoir, China

**DOI:** 10.3390/ijerph16173071

**Published:** 2019-08-23

**Authors:** Shuailong Wen, Tao Wu, Jie Yang, Xue Jiang, Jicheng Zhong

**Affiliations:** 1 State Key Laboratory of Lake Science and Environment, Nanjing Institute of Geography and Limnology, Chinese Academy of Sciences, Nanjing 210008, China; 2 College of Resources and Environment, University of Chinese Academy of Sciences, Beijing 100049, China; 3 Tianjin Hydraulic Research Institute, Tianjin 300061, China

**Keywords:** nutrient, pore water, flux, sediment-water interface, Yuqiao Reservoir

## Abstract

Nutrients released from sediments have a significant influence on the water quality in eutrophic lakes and reservoirs. To clarify the internal nutrient load and provide reference for eutrophication control in Yuqiao Reservoir, a drinking water source reservoir in China, pore water profiles and sediment core incubation experiments were conducted. The nutrients in the water (soluble reactive P (SRP), nitrate-N (NO_3_^−^-N), nitrite-N (NO_2_^−^-N), and ammonium-N (NH_4_^+^-N)) and in the sediments (total N (TN), total P (TP) and total organic carbon (TOC)) were quantified. The results show that NH_4_^+^-N was the main component of inorganic N in the pore water. NH_4_^+^-N and SRP were higher in the pore water than in the overlying water, and the concentration gradient indicated a diffusion potential from the sediment to the overlying water. The NH_4_^+^-N, NO_3_^−^-N, and SRP fluxes showed significant differences amongst the seasons. The NH_4_^+^-N and SRP fluxes were significantly higher in the summer than in other seasons, while NO_3_^−^-N was higher in the autumn. The sediment generally acted as a source of NH_4_^+^-N and SRP and as a sink for NO_3_^−^-N and NO_2_^−^-N. The sediments release 1133.15 and 92.46 tons of N and P, respectively, to the overlying water each year.

## 1. Introduction

Eutrophication is widespread worldwide because of the coupled relationship between ever-growing human population and the concomitant increase in anthropogenic nutrient loading of aquatic ecosystems [1]. The increased nutrient (N and P) loading in overlying water promotes the growth of harmful algae and aggravates algal blooms [2,3]. These pose a threat to freshwater ecosystems and drinking water supplies because cyanobacteria synthesize toxic secondary metabolites, such as cyanotoxins [4]. Controlling the external input is one of the most effective ways to reduce the risk of algal blooms; however, in eutrophic lakes and reservoirs, nutrient release from sediments is also an important source for the overlying water [5,6]. Therefore, controlling and reducing internal nutrient loading that is stored in the sediment is crucial for water quality.

The sediment-water interface (SWI) is one of the most important boundaries in shallow water bodies, such as shallow lakes and reservoirs and coastal and estuarine waters [7]. Nutrient flux at the SWI is a crucial factor affecting nutrient balance and regulating primary productivity in the water [8]. Understanding the mechanisms of N and P cycling at the SWI and the factors controlling nutrients and their fluxes within lakes and reservoirs is essential to enable the authorities take effective decisions pertaining to aquatic ecosystem management and restoration.

The sediment nutrient release rate was estimated via two commonly used methods: (1) sediment core incubation and (2) regression modeling, in combination with concentration gradient diffusion across the SWI. Nutrient flux caused by diffusion in estuaries and coastal areas has been quantified in many studies [9,10,11]. However, Zhang et al. (2013) found that the diffusive and incubated fluxes of dissolved inorganic nitrogen (DIN: NH_4_^+^-N, NO_3_^−^-N, and NO_2_^−^-N) was anisotropic, which indicates that the concentration gradient is not the only factor influencing nutrient flux [12]. In fact, assessing fluxes by concentration gradients is obviously limited as this method ignores the influence of bioturbation by benthic macrofauna and irrigation in the sediment, which could enlarge the flux of SRP and NH_4_^+^-N [12,13]. Although the method of sediment core incubation cannot reflect the physical conditions of lakes such as horizontal flow and other physical factors, it more closely replicates the actual situation in theory because of the impact of bioturbation and other factors, especially in shallow lakes and reservoirs with strong bioturbation [14].

Yuqiao Reservoir is a large drinking water source for Tianjin City, the water quality of which has deteriorated in recent years owing to cyanobacterial blooms occurring during the summer, particularly in 2016. To protect the water quality of Yuqiao Reservoir, the Tianjin Municipal Government has taken a series of measures to control the external input, including relocating polluting enterprises, transferring residents around the reservoir area, banning cage culture, and restoring the ecosystem of the reservoir. However, no corresponding measures have been taken for the internal load of Yuqiao Reservoir. The main reason is that the information on the internal load of Yuqiao Reservoir is lacking, and no research has been conducted to estimate it. The primary objectives of this study were to investigate the temporal and spatial distributions of nutrients in pore water across the SWI, assess the nutrient flux at the SWI during each season, and evaluate the influencing factors in Yuqiao Reservoir. By estimating the internal pollution, information on the internal load of Yuqiao Reservoir was revealed, which can provide a basis for controlling eutrophication. Additionally, this study provides useful information for restoration and management of aquatic ecosystems in similar areas.

## 2. Materials and Methods 

### 2.1. Site Description

Yuqiao Reservoir is located in the east of Jixian County, Tianjin Municipality, China. It is a large-scale water conservancy facility for the purposes of flood control, urban water supply, irrigation, and power generation. The Sha River, Li River, and Lin River are the main rivers that drain into this reservoir. The control catchment of Yuqiao Reservoir is 2060 km^2^, and the average water depth is 3.9 m. Since 1983, Yuqiao Reservoir has been the only source of drinking water, as well as water for industrial and agricultural consumption in Tianjin City. The city has a population of 15.57 million people as per the 2017 data of National Bureau of Statistics. In recent years, with the rapid development of the society and economy around the basin and reservoir area, the reservoir has become seriously polluted by anthropogenic activities. A large number of fish ponds, villages, and farmlands are located around the reservoir, resulting in considerable water pollution and eutrophication. Enrichment of persistent pollutants and other nutrients deteriorate water quality and have an adverse impact on the water supply to Tianjin City [15].

### 2.2. Field Sampling

**Figure 1:**
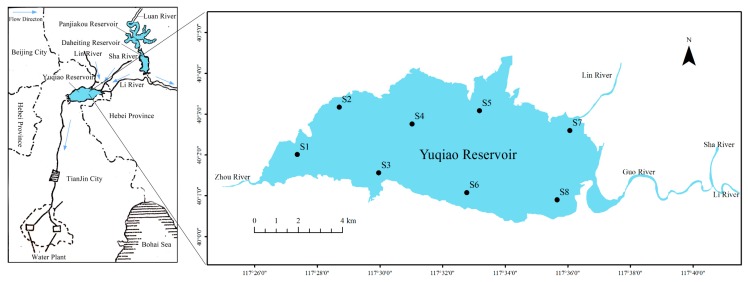
Location map showing the Yuqiao Reservoir and sampling sites.

In this study, eight representative sampling sites were chosen around the reservoir (Figure 1). Surface sediments and overlying water samples were collected in June 2016 for the determination of physical and chemical properties. At each site (S1–S8), three sediment cores were sampled using a gravity corer (90 mm diameter × 500 mm length) in August (summer) and November (autumn) of 2016, and February (winter) and April (spring) of 2017 to measure the flux at the SWI. The collected sediment cores were generally more than 25 cm long and were covered with the near-bottom water from the same location. The core was closed with rubber stoppers to avoid sediment oxidation during the sampling and transportation processes. In addition, 10 L of overlying water was collected in situ and particulate matter was filtered out.

While collecting the sediment core samples, a modified equilibration dialysis device (peeper) was applied to sample the pore water in the sediments. This plastic assembly has a total length of 50 cm with 36 chambers; the distance between the chambers is 0.5 cm, and therefore, the resolution of the pore water we obtained is 1 cm. The theory of the peeper and the sampling methodology involved are described in detail in Johnston et al. (2009) and Xu et al. (2012) [16,17]. Each chamber of the peeper was equipped with anaerobic deionized water, and the peeper was transported to the field site in an anaerobic environment. The peepers were inserted vertically into the sediment of the reservoir, leaving a part of the peeper in the overlying water above the sediment so that a complete SWI profile could be obtained. The peepers were taken out after 15 days for ion-exchange equilibrium. By that time, the concentrations of DIN and SRP in the pore water were the same as those in each chamber of the peeper. After retrieval of the peepers, the pore water of the peepers was sampled immediately, injected into vials containing appropriate fixative agents, and stored on ice for further processing.

### 2.3. Incubation of Sediment Cores 

To study the potential exchange of nutrients at the SWI, three replicates of sediment cores from each site were incubated in a water tank in the dark at the in-situ water temperature (±2 °C). The siphon method was used to remove the overlying water from the sediment cores just sampled, and then filtered in-situ water was carefully poured along the sediment column walls to avoid disturbance, and the depth of the overlying water was maintained at 20 cm. Water samples (50 mL), taken from the incubated cores at designated intervals (0, 12, 24, 36, 48, 60, and 72 h) from the location of 5 cm above the SWI, were filtered through 0.45 μm syringe filters and analyzed for SRP and DIN. After sampling, the same volume of the original filtered water was added to each core immediately to maintain the water quantity. The diffusion flux of nutrients at SWI can be estimated based on the changes of concentration in the overlying water in the system.

Nutrients fluxes across the SWI were estimated by the static release method [18,19]:

(1) $$F=\left[V\left(C_{n}-C_{0}\right)+\sum_{j=1}^{n}V_{j-1}\left(C_{j-1}-C_{a}\right)\right]/\left(S\times t\right),$$

where F is the diffusive flux across the SWI in mg·m^−2^·day^−1^; V is the water volume in the sediment core in L; C_n_, C_0_, and C_j-1_ are the nutrient concentrations in n times, 0 (initial) and j-1 times in mg·L^−1^, respectively; C_a_ is the nutrient concentration in the water that was added to the sediment core after each sampling time in mg·L^−1^; V_j-1_ is the volume of water sampled from the sediment core in L; S is the area across the SWI in the sediment core in m^2^; and t is the incubation time in d. The calculated diffusion fluxes of nutrients across the SWI represent the average exchange fluxes over three days.

### 2.4. Sample Analysis

The water content of the surface sediment is determined by drying fresh sediment to a constant weight at 105 °C. TN and TP in the sediments were determined by the potassium persulfate oxidation method, while TOC was determined by the potassium dichromate oxidation-ferrous sulfate titration method [20]. The concentration of TN in water was measured by the alkaline potassium persulfate digestion-UV spectrophotometric method. TP was determined using the molybdenum blue method, following digestion with alkaline potassium persulfate. The concentrations of DIN and SRP in water were measured spectrophotometrically with a Skalar Nutrient Analyzer (Skalar San ++, Breda, Netherlands), according to the methods described by Grasshoff et al. (1984) [21].

### 2.5. Statistical Analysis

The samples in the experiments were measured in duplicate, and the results are presented as means (±SD). Graphic plots were obtained using OriginPro 2017C software (Originlab, Northampton, MA, USA). Significant differences between the seasons were identified through analysis of variance (ANOVA) followed by the Tukey’s test. Excel 2013 (Microsoft, Redmond, WA, USA) and SPSS (IBM SPSS Statistics 22.0 for Windows, SPSS Inc., Chicago, IL, USA) were used for quantitative analyses of data.

## 3. Results

### 3.1. Nutrients in the Surface Sediment and Water

The contents of TN and TP in the surface sediments were 2023.53–3880.25 and 532.88–817.74 mg/kg, respectively, while the contents of TOC in the sediments were 1.84–4.68% (Table 1). In the surface water, the concentrations of TN and TP were 2.13–4.11 and 0.18–0.40 mg/L, respectively. The concentrations of NH_4_^+^-N, NO_3_^−^-N, NO_2_^−^-N, and SRP in the surface water were 0.36–0.54, 0.38–0.72, 0.003–0.007, and 0.001–0.09 mg/L, respectively. In the surface water, DIN accounted for 26.92–43.50% of the TN in the surface water, while SRP accounted for 0.58–35.23% of the TP. NH_4_^+^-N and NO_3_^−^-N were the main components of DIN in the surface water, which accounted for 34.50–58.73% and 40.55–65.02%, respectively.

### 3.2. DIN and SRP Profiles at the SWI

**Figure 2:**
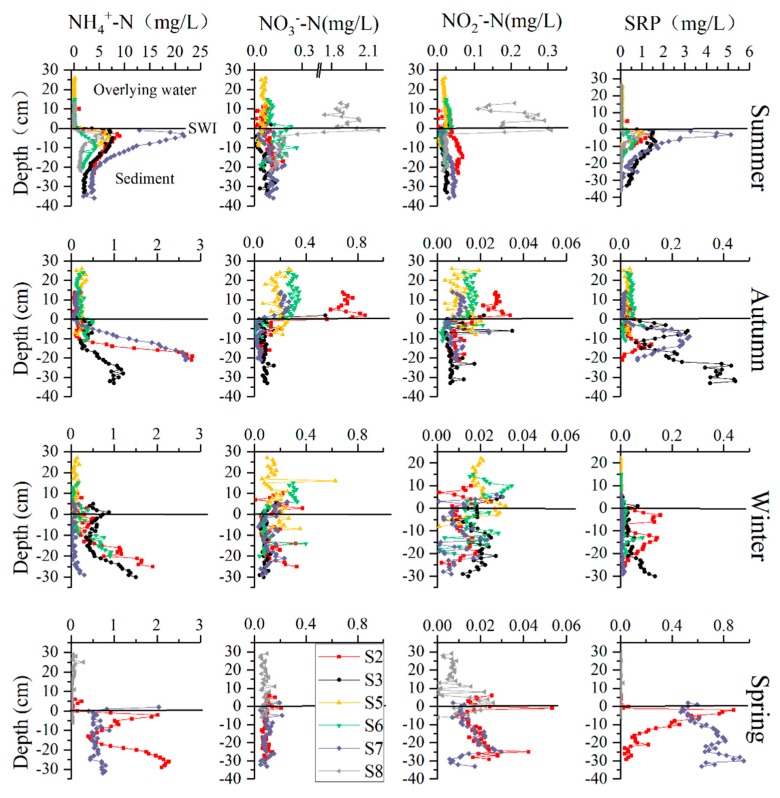
Nutrient concentrations in overlying water and pore water at the sediment-water interface (SWI). Vertical profiles of nutrients at SWI were not obtained at some sites because of difficulties in collecting the samples at the sites or some peepers which had been inserted in the sediments were lost.

The nutrient profiles at the SWI are illustrated in Figure 2. No significant difference was observed in NH_4_^+^-N concentrations in the overlying water above the SWI during each season. NH_4_^+^-N concentrations increased with depth from the SWI to a depth of −4 cm, and then decreased with depth at all sites in the summer. Maximum concentrations of NH_4_^+^-N in the pore water at S2, S3, S5, S6, S7, and S8 were 9.01, 7.59, 6.89, 4.28, 21.78, and 5.69 mg/L respectively. NH_4_^+^-N concentrations increased with depth at all sites in the autumn and at sites S2, S5, S6, and S7 in the winter. In contrast, NH_4_^+^-N concentrations decreased with depth from the SWI to a depth of −10 cm and then increased with depth at site S3 in the winter. In spring, the NH_4_^+^-N concentrations at S8 were low, and no significant vertical change was noted below the SWI, while NH_4_^+^-N changed slightly with depth at S7. Interestingly, NH_4_^+^-N concentrations increased with depth and then decreased, and then repeated the above pattern at S2.

Concentration of NO_3_^−^-N in the overlying water was significantly higher than that in the pore water at S8 in summer. The NO_3_^−^-N concentration in the overlying water was 1.91 mg/L, which then decreased significantly at the SWI, where the NO_3_^−^-N concentration in the pore water was 0.39 mg/L. There was no significant difference in NO_3_^−^-N concentrations between the overlying water and pore water in summer, except at S8. The average NO_3_^−^-N concentrations in the overlying water and pore water were 0.07 and 0.09 mg/L, respectively, with slight vertical fluctuations. In autumn, the NO_3_^−^-N concentrations in the overlying water were higher than those in the pore water, where the average concentrations were 0.36 and 0.09 mg/L, respectively. Variation in the NO_3_^−^-N concentrations were observed at the SWI along with slight vertical fluctuations, with average concentrations of 0.14 and 0.09 mg/L in winter and spring, respectively. The spatial and temporal distribution pattern of NO_2_^−^-N was found to be very similar to that of NO_3_^−^-N; the average concentrations of NO_2_^−^-N at the SWI were 0.04, 0.01, 0.02, and 0.02 mg/L in summer, autumn, winter, and spring, respectively.

The SRP concentrations in the summer were low in the overlying water, and increased with depth from the SWI to −5 cm and then decreased with depth. The maximum concentration occurred at S7, while the minimum occurred at S8. In contrast to the pattern in the summer, the SRP concentrations in the pore water in autumn, winter, and spring, increased first and then decreased with depth several times, such as at S3 in the autumn, S2 in the winter, and S7 in the spring. The SRP concentrations in the pore water were highest in the summer, followed by spring and autumn, and lowest in the winter, with values ranging between 0.003–5.13, 0.001–0.96, 0.008–0.45, and 0.00–0.15 mg/L, respectively.

NH_4_^+^-N was the main form of DIN in the pore water, and the concentrations of NH_4_^+^-N and SRP in the pore water were significantly higher in the summer than in other seasons. In terms of spatial distribution, NH_4_^+^-N and SRP in the pore water in the summer showed a similar pattern with S7 > S2 > S3 > S5 > S8 > S6 and S7 > S3 > S2 > S5 > S8 > S6, respectively. Overall, NH_4_^+^-N and SRP in the pore water showed a similar spatial distribution, which was higher in the Lin Estuary and downstream of the reservoir and lower at S8 and midstream in the reservoir. The concentrations of NH_4_^+^-N and SRP in spring and autumn showed similar spatial patterns to that in the summer, but in winter, NH_4_^+^-N and SRP in the pore water were low, particularly at S7.

### 3.3. Fluxes of DIN and SRP at the SWI

**Figure 3:**
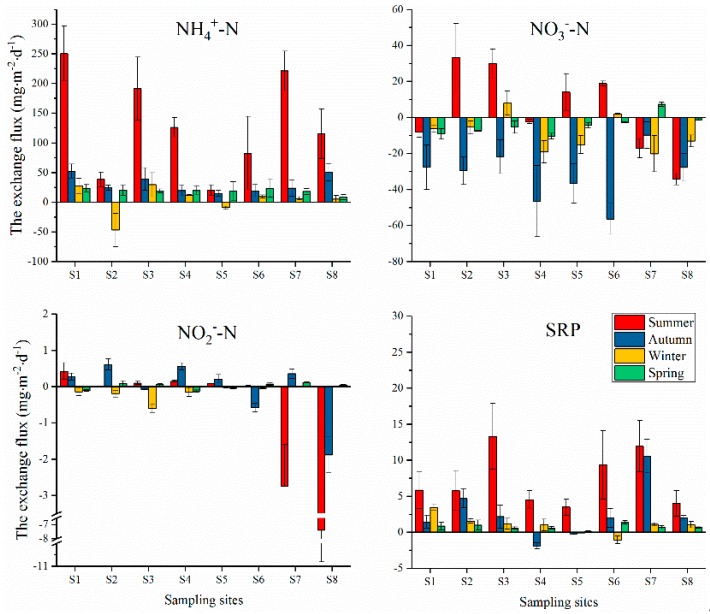
Diffusive fluxes of inorganic nitrogen and phosphate at the SWI. Positive fluxes indicate diffusion out of the sediments.

**Figure 4:**
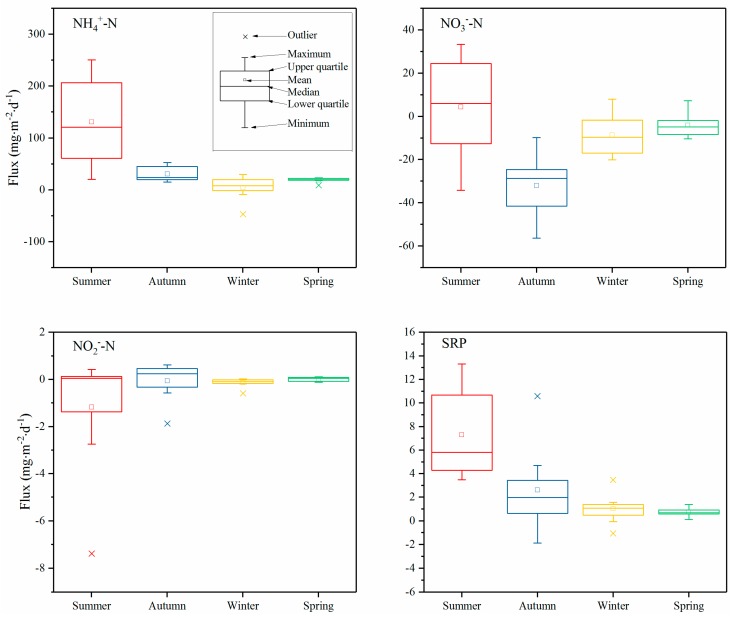
Diffusive fluxes of nutrients across the SWI in different seasons.

Fluxes of NH_4_^+^-N, NO_3_^−^-N, NO_2_^−^-N, and SRP at the SWI are given in Figure 3. For NH_4_^+^-N, the sediment acted as a N source for the water column throughout the year, except at sites S2 and S5 in the winter. The NH_4_^+^-N fluxes in the summer were significantly higher than in other seasons (*p* < 0.01, Figure 4), with the highest value at S1 (250.52 ± 46.52 mg·m^−2^·day^−1^), lowest value at S5 (20.60 ± 8.73 mg·m^−2^·day^−1^), and with an average value of 130.98 ± 84.03 mg·m^−2^·day^−1^. The diffusion fluxes in autumn, winter, and spring were relatively low, with mean values of 30.57 ± 14.79, 4.38 ± 24.09, and 19.00 ± 4.63 mg·m^−2^·day^−1^, respectively. The seasonal differences in autumn, winter, and spring were not significant (*p* > 0.05).

NO_3_^−^-N was transferred from the overlying water to the sediment, where the latter played the role of a sink at almost all sites throughout the year, except at a few sites in the summer. The NO_3_^−^-N fluxes transferred to the sediment were higher in the autumn than in winter and spring. In the summer, the NO_3_^−^-N was released from the sediment to the overlying water at S2, S3, S5, and S6, while it showed the opposite trend at S1, S4, S7, and S8. The mean value of the fluxes of NO_3_^−^-N in summer, autumn, winter, and spring were 4.35 ± 23.78, −31.96 ± 14.46, −8.60 ± 10.03, and −4.13 ± 5.58 mg·m^−2^·day^−1^, respectively. The fluxes showed a significant difference between autumn and the other seasons (*p* < 0.05, Figure 4).

The NO_2_^−^-N diffusion fluxes showed great heterogeneity in terms of space (Figure. 3). The NO_2_^−^-N transferred from the overlying water to the sediment with higher values at S7 and S8 in the summer (−2.74 ± 1.16 and −7.38 ± 3.44 mg·m^−2^·day^−1^, respectively) and at S8 in the autumn (−1.88 ± 0.49 mg·m^−2^·day^−1^). At other sites, the NO_2_^−^-N fluxes were generally low, and no distinct seasonal differences were observed. The NO_2_^−^-N fluxes in summer, autumn, winter, and spring were −1.17 ± 2.71, −0.06 ± 0.83, −0.14 ± 0.20, and 0.01 ± 0.09 mg·m^−2^·day^−1^, respectively, with no significant differences among the seasons (*p* > 0.05). From the above, we can see that NH_4_^+^-N was the main exchange form of inorganic N across the SWI, and the NH_4_^+^-N, NO_3_^−^-N_,_ and NO_2_^−^-N fluxes as a fraction of DIN were 77.07 ± 12.93%, 22.14 ± 12.73%, and 0.79 ± 1.06%, respectively.

SRP was released from the sediment to the overlying water at all sites throughout the year, except at S4 in the autumn and S6 in the winter, such that the sediment acted as a source of P, similarly to N. The highest and lowest fluxes of SRP in the summer were at S3 (13.31 ± 4.59 mg·m^−2^·day^−1^) and S5 (3.50 ± 1.14 mg·m^−2^·day^−1^), respectively. The mean value of SRP was 7.29 ± 3.76 mg·m^−2^·day^−1^, followed by the fluxes in autumn, winter, and spring, which were 2.61 ± 3.75, 1.05 ± 1.30, and 0.74 ± 0.36 mg·m^−2^·day^−1^, respectively. It is obvious that the fluxes in the summer were higher than those in other seasons (*p* < 0.05), but there were no significant differences between autumn, winter, and spring (*p* > 0.05).

## 4. Discussion

### 4.1. Characteristics of the Surface Sediments

The sediment, especially the surface sediment (0–5 cm), can greatly influence the water quality, especially that of overlying water [22]. The TN and TP in the sediment in Yuqiao Reservoir were considerably higher than that in other eutrophic lakes, such as Lake Chivero [23], Lake Taihu [24], and Jinpen Reservoir [25]. The correlation between TN and TOC was significant (two-tailed test, r = 0.949, *p* < 0.01), a result which shows that organic matter was the main source of N. In contrast, TP and TOC had no similar correlation (r = 0.600, *p* = 0.116), which indicates different sources for TN and TP, where the sources for TP in the sediments may be influenced by other factors [26]. N and C in Yuqiao Reservoir might be mainly derived from aquaculture feed, while mineral exploitation associated with mining and runoff around the reservoir might be the major sources of P. 

### 4.2. Characteristics of Nutrients in the Pore Water

Generally, N and P that have been adsorbed by sediments enter the pore water first when they diffuse across the SWI. This step is often considered as a determinant process in the diffusion of N and P; these elements then mix and diffuse to the upper multiphase interface and the overlying water, while the intensity of the diffusion mainly depends on the concentration gradient of these nutrients in the pore water of the sediments [27]. NH_4_^+^-N and SRP in pore water were significantly higher in the summer than in other seasons. Concentrations of NH_4_^+^-N in summer, autumn, winter, and spring were 0.17–21.78, 0.05–2.82, 0.01–1.89, and 0.01–2.26 mg/L, respectively, while those of SRP were 0.01–5.13, 0.01–0.45, 0.01–0.15, and 0.01–0.96 mg/L, respectively. Most often, sedimentary organic matter is mineralized at the fastest rate in summer and this promotes the production of NH_4_^+^-N and SRP [28]. Spatial distribution of these nutrients in the pore water is quite variable and is related to the location conditions, dissolved oxygen (DO), microbial activity, and disturbance. S7 is near the Lin Estuary with a high number of nearby residential areas. Pollutants discharged by human activities enter the reservoir, which renders the pollution relatively serious. At the same time, owing to the deposition of pollutants and the accumulation of algae on the surface of the sediments, the pollution downstream of the reservoir is also serious. The decomposition of organic matter accumulated in the sediments leads to changes in the DO and pH of these sediments and increases their C, N, and P contents [29]; this might be the reason for the high N and P contents in the pore water in the area.

In contrast to the overlying water, DIN in the pore water was dominated by NH_4_^+^-N and also featured high NH_4_^+^-N and low NO_2_^−^-N. The early diagenesis of organic matter and nitrification/denitrification results in different NH_4_^+^-N, NO_3_^−^-N, and NO_2_^−^-N contents in the pore water [12,30]. Organic N in the sediment can be mineralized into a large amount of NH_4_^+^-N via the action of microorganisms. NH_4_^+^-N was the main form of DIN in the pore water (94.92%) in this study. NH_4_^+^-N is stored in the pore water or adsorbed onto the surfaces of sediment minerals in an anaerobic environment, such that the release of NH_4_^+^-N from the pore water is an important source of N in the overlying water. Generally, NO_3_^−^-N and NO_2_^−^-N concentrations are relatively high in the overlying water; these diffuse into the sediments through the SWI due to a variety of driving factors, such as concentration gradients and DO [12]. Our research revealed similar observations at most of the sites; this was also confirmed by the diffusion fluxes of NO_3_^−^-N and NO_2_^−^-N at the SWI.

Regarding P species in the sediments of Yuqiao Reservoir, Jiang et al. (2018) found that Fe-bound P was the most abundant of the latent active forms of P, while the content of organic P in the reservoir was generally low [31]. SRP is generally higher in the pore water than in the overlying water, and the concentration gradient indicates that SRP mainly diffuses from the pore water to the overlying water, with the sediment acting as a source. The main reason for this is that in anoxic sediments, Fe-bound P and P-containing organic matter that is easily decomposed can release free SRP through reduction of ferric iron and decomposition of organic matter, respectively [32,33].

### 4.3. Nutrient Fluxes at the SWI

Increase in microbial reactions with temperature is capable of enhancing the molecular diffusion rate, while the reduction of NO_3_^−^-N to NH_4_^+^-N could be the result of both denitrification and dissimilatory NO_3_^−^-N reduction that releases NH_4_^+^-N into the sediment pore water; the latter rapidly diffuses along the concentration gradient towards the overlying water [28,34]. The fluxes of NH_4_^+^-N across the SWI in Yuqiao Reservoir were higher than those in Lake Taihu and Lake Nansihu in the summer, higher than that in Lake Taihu in the winter, and lower than that in Lake Dianchi and Zhoucun Reservoir in the autumn (Table 2). The main reasons for this observation were as follows: (1) the N content in the sediments of Yuqiao Reservoir was higher than that in Taihu Lake and Nansihu Lake [35,36], and (2) the NH_4_^+^-N concentration gradient between pore water and overlying water of Yuqaio Reservoir was larger than that of Taihu Lake and Nansihu Lake in the summer, and smaller than that of Dianchi Lake and Zhoucun Reservoir in the autumn [19,37]. N content in sediment and NH_4_^+^-N concentration in pore water are the key factors affecting NH_4_^+^-N release to overlying water [27]. The release flux of NH_4_^+^-N in Yuqiao Reservoir is higher in the summer than in the other three seasons (Figure 4). Bowden (1984) and Rocha (1998) also found that an increase in temperature was favorable for the release of NH_4_^+^-N from the sediments, mainly because the temperature rise can improve the ammoniation rate of organic matter in the sediments [38,39]. The average temperature in Yuqiao Reservoir is higher than 25 °C in summer, and decomposition of cyanobacteria consumes a large amount of DO, while the sediments are in either anoxic or anaerobic conditions, which promotes the release of NH_4_^+^-N and SRP [40,41,42]. Yuqiao Reservoir is located in the temperate monsoon zone, and the surface runoff caused by frequent rainfall in the summer leads to a large amount of organic matter entering the reservoir. Organic N in the sediment is much higher than inorganic N in Yuqiao Reservoir, and with degradation of the organic matter, NH_4_^+^-N is transferred into the pore water and diffuses to the overlying water owing to the concentration gradient. 

The diffusion rate of NO_3_^−^-N showed a significant difference amongst the four seasons (Figure 4). The sediment acted as a source of NO_3_^−^-N in the summer. However, NO_3_^−^-N was transferred from the overlying water to the pore water, where the sediment played the role of a sink in autumn, winter, and spring. For NO_2_^−^-N, the flux represented a relatively small fraction of the inorganic N, and the sediment played the role of a sink. NH_4_^+^-N and NO_3_^−^-N were the main forms of inorganic N exchange at the SWI. The differences in the flux of inorganic N exchange caused by NO_2_^−^-N were negligible; however, these species are still important as intermediates of nitrification and denitrification processes [12]. In the N cycle, the transport and transition of N at the SWI is complex [41]. In fact, concentration gradient, temperature, DO, bioturbation, porosity, nitrification, denitrification, and ammoniation collectively influence the reactions at the SWI [12,41,43,44]. Lower redox potentials and oxygen depletion rates in the sediment enhance ammonification, that promote the release of NH_4_^+^-N [45]. Under oxic conditions, nitrification can readily occur and NO_3_^−^-N concentrations in the sediment can increase, causing NO_3_^−^-N to diffuse from the sediment to the overlying water [41,46]. However, nitrification is restricted, and denitrification occurs under reducing conditions, such that NO_3_^−^-N is reduced and NO_2_^−^-N and N_2_ are generated [47]. Morse et al. (2005) found that the redox conditions affect the adsorption of NH_4_^+^-N in the sediments under anoxic conditions; NH_4_^+^-N is more easily desorbed and diffuses from the pore water to the overlying water [48]. In addition, suitable temperatures during the summer promote the activity of bottom fauna, and bioturbation enhances the release of NH_4_^+^-N from the sediments [13].

The release flux of SRP in the summer was higher than that in the autumn and spring and was lowest in the winter (Figure 4). The SRP flux across the SWI in summer was approximately three times that in the autumn and almost an order of magnitude greater than in the winter. The release flux of SRP at Yuqiao Reservoir was much higher compared with other lakes and reservoirs, such as Lake Taihu, Lake Dianchi, and Zhoucun Reservoir (Table 2). Higher temperatures and lower DO may accelerate the diffusion of SRP [49,50]. Kang et al. (2018) found that under anoxic conditions, polyphosphate degrades and releases orthophosphate [51]. Under anaerobic conditions, Fe^3+^ can be reduced to Fe^2+^, and SRP can be adsorbed by iron/manganese released into the pore water along with iron hydroxide dissolution [12]. In summer, DO plays an important role in P exchange between the water and sediments [40,42]. In our study, with the anoxic conditions at the SWI in the summer, SRP was more easily released from the sediments to the overlying water. In fact, previous studies have shown that the aerobic layer at the millimeter level on the surface of the sediments and the bottom diffusion boundary layer across the SWI prevent the migration of SRP from the pore water into the overlying water [27,30,52]. However, when DO decreases at the SWI, the aerobic layer or diffusion boundary layer becomes thinner or disappears, and SRP in the pore water can diffuse more readily to the overlying water with the concentration gradient [52,53]. In an oxidized state, NO_3_^−^-N acts as an alternative electron acceptor that can suppresses the reduction of Fe^3+^, preventing the release of Fe-bound P in the sediments [51,54]. The smaller flux of SRP across the SWI during the winter was most likely due to the binding of phosphate to oxidized iron, which is more abundant within the sediments during the cold season, when oxygen demand is lower and oxygen penetration into the sediments is deeper [55,56].

In terms of their spatial pattern, the NH_4_^+^-N and SRP fluxes were higher in the Lin River Estuary (S7) and the downstream of the reservoir in the summer (except the NH_4_^+^-N flux at S2) but were lower at S8 and the midstream of the reservoir. This is basically consistent with the distribution pattern of NH_4_^+^-N and SRP in the pore water. Higher concentrations of NH_4_^+^-N and SRP in the pore water at S3 and S7 in the summer were accompanied by higher release fluxes. Fresh organic matter, such as dead algae residue, often accumulates downstream of the reservoir. The Lin River Estuary is greatly affected by human activities, such that more pollutants get concentrated in these areas. The pollutants accumulated in the sediments of Yuqiao Reservoir were often rich in organic N and P because of net-pen fish culture and anthropogenic emissions. Tyler et al. (2003) reported that the unstable organic N and P at the SWI have higher decomposition rates [57], while the rapid mineralization of organic matter in the surface sediments and release of nutrients is one of the main ways of releasing internal N and P [58]. Different physical, chemical, and biological environments lead to different nutrient release potentials, resulting in different spatial patterns in the internal releases.

### 4.4. Estimation of Annual DIN and SRP Fluxes in Yuqiao Reservoir

Based on the DIN and SRP exchange fluxes at the SWI in the four seasons and the area of Yuqiao Reservoir (86.7 km^2^), we can estimate the annual DIN and SRP release fluxes at the SWI of this reservoir. The annual average release fluxes of NH_4_^+^-N, NO_3_^−^-N, NO_2_^−^-N, and SRP at the SWI were 46.23, −10.08, −0.34, and 2.92 mg·m^−2^·day^−1^, respectively; the release fluxes in Yuqiao Reservoir were 1463.00, −319.11, −10.73, and 92.46 t/a, respectively. The sediment is the source of NH_4_^+^-N and SRP, while it acted as a sink for NO_3_^−^-N and NO_2_^−^-N. Overall, the sediment released 1133.15 tons of DIN and 92.46 tons of SRP to the overlying water. Internal loading is an important cause of eutrophication in Yuqiao Reservoir.

### 4.5. Countermeasures and Suggestions for Eutrophication Control of Yuqiao Reservoir

Since 2015, Tianjin City and Hebei Province have invested hundreds of millions of yuan to prevent and control the pollution of the upstream water of Yuqaio Reservoir, including setting up a pre-reservoir before the inlet estuary of Yuqiao Reservoir for pollution interception. Net cage culture of Panjiakou and Daheiting Reservoirs in the upper reaches was banned, and residents in the south bank of Yuqiao Reservoir were relocated. All these measures proved useful in controlling the external pollution of Yuqaio Reservoir. However, the results of our study demonstrated that the release of internal N and P from the sediment cannot be ignored. Therefore, in view of the internal pollution, the commonly used remediation methods including in-situ capping, algae salvaging, ecological remediation, and sediment dredging can be used for eutrophication control of Yuqiao Reservoir. For areas with large internal release flux, such as S1, S3, and S7 (Figure 3), dredging can be used to remove internal pollutants. For areas with relatively small flux, ecological restoration can be implemented. However, considering that there are still residential areas in the north bank of the reservoir that significantly impact water quality, further strengthening the control of the external pollution and realizing the integrated management of the internal and external sources can effectively restore the water quality of this eutrophic reservoir.

## 5. Conclusions

The following major conclusions can be drawn from this study:

1. High TN, TP, and TOC contents occurred in the sediments at Yuqiao Reservoir compared with other eutrophic lakes and reservoirs. Concentrations of NH_4_^+^-N and SRP in the pore water are generally higher than those in the overlying water in contrast to those of NO_3_^−^-N and NO_2_^−^-N. NH_4_^+^-N was the main form of DIN in the pore water.
2. The fluxes of NH_4_^+^-N, NO_3_^−^-N, NO_2_^−^-N, and SRP indicate that the sediment was the source of NH_4_^+^-N and SRP, while it was a sink for NO_3_^−^-N and NO_2_^−^-N. The fluxes of NH_4_^+^-N, NO_3_^−^-N, and SRP were higher in the summer compared to other seasons. At the annual scale, the sediment released 1133.15 and 92.46 tons of N and P, respectively, to the overlying water, which indicates that the internal release of N and P plays an important role in maintaining the eutrophic status of Yuqiao Reservoir. Technical measures such as sediment dredging should be adopted to control the internal load.

## Figures and Tables

**Table 1:** Physicochemical characteristics of surface sediments and water columns.

Site	Sediment				Surface Water					
	TN	TP	TOC	Water Content	TN	TP	NH_4_^+^-N	NO_3_^−^-N	NO_2_^−^-N	SRP
	mg/kg	mg/kg	%	%	mg/L	mg/L	mg/L	mg/L	mg/L	mg/L
S1	3208.72	555.69	3.04	74.62	3.55	0.40	0.43	0.61	0.005	0.07
S2	3521.97	578.51	3.38	73.92	2.13	0.27	0.54	0.38	0.007	0.09
S3	3763.91	599.87	4.27	79.41	2.73	0.19	0.39	0.44	0.006	0.00 ^1^
S4	3880.25	817.74	4.68	80.86	4.11	0.34	0.38	0.72	0.005	0.06
S5	2507.14	630.17	2.90	65.84	2.57	0.24	0.42	0.47	0.003	0.03
S6	2023.53	639.90	1.84	68.79	2.67	0.20	0.49	0.50	0.004	0.01
S7	2538.69	552.71	2.46	59.96	2.28	0.18	0.36	0.42	0.005	0.01
S8	2179.71	532.88	2.19	65.64	3.68	0.31	0.49	0.59	0.004	0.03

^1^ Below detection limit. TN = total N. TP = total P. TOC = total organic carbon. SRP = soluble reactive P.

**Table 2:** Nutrient fluxes in previously published studies across the sediment-water interface.

Source	Location	Season	NH_4_^+^-N	NO_3_^−^-N	NO_2_^−^-N	SRP	Method
			(mg·m^−2^·Day^−1^)	(mg·m^−2^·Day^−1^)	(mg·m^−2^·Day^−1^)	(mg·m^−2^·Day^−1^)	
Fan et al. 2004 [35]	Lake Taihu, China	Summer	34.1 ± 20.8	—	—	—	Sediment core incubation
		Winter	−16.0 ± 17.6	—	—	—	
Li et al. 2008 [19]	Lake Dianchi, China	Autumn	22.94–163.12	—	—	0.90–2.06	Sediment core incubation
Wang et al. 2013 [36]	Lake Nansihu, China	Summer	3.1–10.3	—	—	0.3–2.7	Sediment core incubation
Petranich et al. 2018 [34]	Marano and Grado Lagoon, Italy	Summer	24.3	22.32	0.874	2.28	In-situ benthic chambers
		Autumn	10.98	32.24	0.506	0.285	
		Winter	3.96	−8.68	−0.138	−0.19	
Denis and Grenz 2003 [30]	Gulf of Lions, NW Mediterranean	Spring	−0.4–3.67	1.61–17.55	−1.7–0.1	−0.64–2.76	Sediment core incubation
Huang et al. 2016 [37]	Zhoucun Reservoir, China	Autumn	62.83–133.23	—	—	0.36–1.27	Diffusive (Fick’s Law)
This study	Yuqiao Reservoir, China	Spring	19.00 ± 4.63	−4.13 ± 5.58	0.01 ± 0.09	0.74 ± 0.36	Sediment core incubation
		Summer	130.98 ± 84.03	4.35 ± 23.78	−1.17 ± 2.71	7.29 ± 3.76	
		Autumn	30.57 ± 14.79	−31.96 ± 14.46	−0.06 ± 0.83	2.61 ± 3.75	
		Winter	4.38 ± 24.09	−8.60 ± 10.03	−0.14 ± 0.20	1.05 ± 1.30	
